# Chemically-Modified Cellulose Paper as a Microstructured Catalytic Reactor

**DOI:** 10.3390/molecules20011495

**Published:** 2015-01-15

**Authors:** Hirotaka Koga, Takuya Kitaoka, Akira Isogai

**Affiliations:** 1 The Institute of Scientific and Industrial Research, Osaka University, 8-1 Mihogaoka, Ibaraki, Osaka 567-0047, Japan; 2 Department of Agro-environmental Sciences, Graduate School of Bioresource and Bioenvironmental Sciences, Kyushu University, 6-10-1 Hakozaki, Higashi-ku, Fukuoka 812-8581, Japan; E-Mail: tkitaoka@agr.kyushu-u.ac.jp; 3 Department of Biomaterials Sciences, Graduate School of Agricultural and Life Sciences, The University of Tokyo, 1-1-1 Yayoi, Bunkyo-ku, Tokyo 113-8657, Japan; E-Mail: aisogai@mail.ecc.u-tokyo.ac.jp

**Keywords:** cellulose paper, microreactor, catalyst, silane coupling

## Abstract

We discuss the successful use of chemically-modified cellulose paper as a microstructured catalytic reactor for the production of useful chemicals. The chemical modification of cellulose paper was achieved using a silane-coupling technique. Amine-modified paper was directly used as a base catalyst for the Knoevenagel condensation reaction. Methacrylate-modified paper was used for the immobilization of lipase and then in nonaqueous transesterification processes. These catalytic paper materials offer high reaction efficiencies and have excellent practical properties. We suggest that the paper-specific interconnected microstructure with pulp fiber networks provides fast mixing of the reactants and efficient transport of the reactants to the catalytically-active sites. This concept is expected to be a promising route to green and sustainable chemistry.

## 1. Introduction

Techniques for the environmentally-benign catalytic conversion of various chemical substances have played essential roles in a wide range of industrial processes, such as the production of useful chemicals, environment purification and energy conversion. The escalation of energy, environmental and resource problems has resulted in an urgent need to develop high-performance catalytic materials that can effectively promote specific chemical reactions. Metal-based catalysts, such as metal nanoparticles [1,2] and metal complexes [3], are promising catalysts for a variety of chemical reactions. However, there are increasing requirements to break the dependence on metal-based catalysts, because of the limited availability of metal resources. To deal with this issue, many researchers have developed various catalytic materials as alternatives to metal-based catalysts, including small organic molecules [4,5] and enzymes [6,7].

The trend toward green and sustainable development has promoted the effective use of renewable bioresources in catalytic applications. Cellulose and chitin are the most common and abundant biopolymers in nature. In previous studies, highly crystalline nanofibrils were extracted from cellulose and chitin, and these have been intensively investigated for a wide range of uses, such as high-strength materials [8,9], gas-barrier films [10], transparent materials [11,12] and flexible electronics [13,14,15,16,17]. In catalytic applications, cellulose nanofibrils have been used as supporting materials for metal nanocatalysts, including nanoparticles [18,19] and ions [20]. Highly-dispersed and exposed metal nanocatalysts on the crystalline surfaces of cellulose nanofibrils give higher catalytic performances compared with conventional polymer-supported metal nanocatalysts, because of the efficient contact with the reactants. Recently, native chitin aerogels with primary C2 amines were directly used as base catalysts for the Knoevenagel condensation reaction [21]. These studies indicate the great potential of cellulose and chitin in catalytic applications.

Much effort has been devoted to improving the activities of catalysts, but structured supports that contain micrometer-scale open paths for fluids are also receiving increasing attention, because they enable fast mixing and effective diffusion of heat and reactants, especially in continuous-flow catalytic reactions, providing high reaction efficiency [22]. Many structured supports, such as microchannels [23] and honeycombs [24,25], have been developed. Typical examples are honeycomb-structured supports with regularly-arranged parallel channels, which have been used for exhaust gas purification. However, they have several disadvantages, including high weights and lack of radial reactant mixing [25]. In addition, the preparation of structured supports with narrow microchannels generally requires complicated, multistep processes, such as laser machining [26] or ice-templating methods [27]. The facile preparation and structural design of porous catalytic supports are therefore significant challenges to establishing more effective catalytic processes.

**Figure 1:**
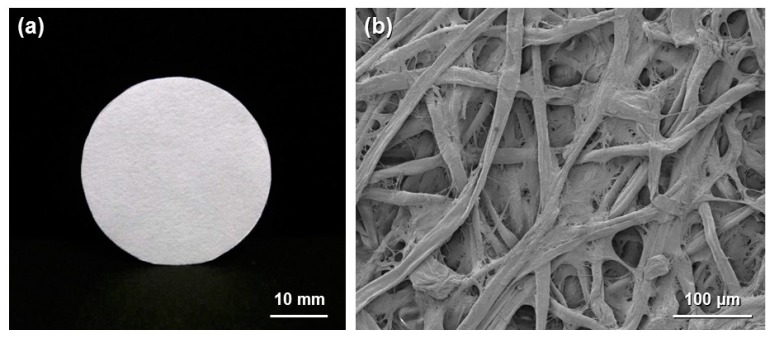
(**a**) Optical image of circular cellulose paper; and (**b**) field-emission scanning electron microscopy image of paper.

Paper is a typical cellulosic material and is traditionally used in daily life for various purposes, such as writing, printing, wiping, wrapping and packaging applications. Paper materials are prepared from cellulose pulp fibers using a high-speed and low-cost papermaking process. The high mass production and excellent practical properties (*i.e.*, low cost, light weight and high flexibility) of paper have resulted in increasing interest in the development of new functionalized paper materials. Cellulose paper has an interconnected porous microstructure derived from layered pulp fiber networks (Figure 1) and can be chemically modified through the OH groups of cellulose to offer catalytic functions. Paper is therefore promising as a structured catalyst support.

In this review, we describe successful applications of cellulose papers as microstructured catalytic reactors for the effective production of useful chemicals. First, we describe the facile and direct introduction of various functional groups into paper using a silane-coupling technique to provide catalytic functions. Secondly, we discuss the catalytic performances of amine-modified paper in the Knoevenagel condensation reaction and lipase immobilized on methacrylate-modified paper in nonaqueous esterification reactions. The effect of the paper-specific porous microstructure on the catalytic reaction efficiency is also reviewed.

## 2. *In Situ* Modification of Cellulose Paper with Functional Groups Using the Silane-Coupling Technique

Silane coupling has frequently been used to functionalize inorganic materials, such as silica and alumina [28,29]. Silane coupling involves a condensation reaction between the OH groups of organofunctional silane-coupling agents and inorganic substrates and is a useful approach for the introduction of various functional groups into target substrates. Cellulose has three OH groups per anhydroglucose unit and can be used in silane-coupling processes. Some researchers have reported the silane-coupling treatment of cellulosic materials, such as the surface modification of cellulose fibers to increase their hydrophobicity [30,31] and grafting of cellulose strands onto the surface of silica [32].

**Figure 2:**
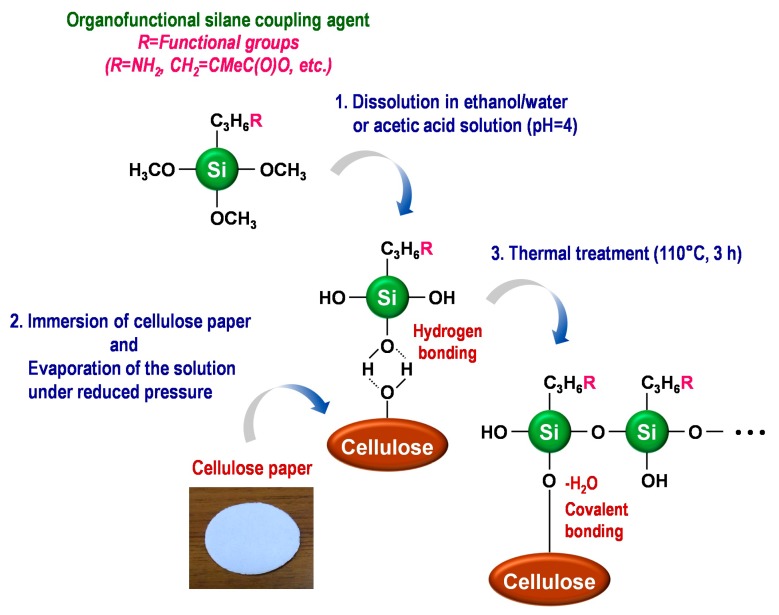
Schematic diagram of the silane-coupling process on cellulose paper.

**Figure 3:**
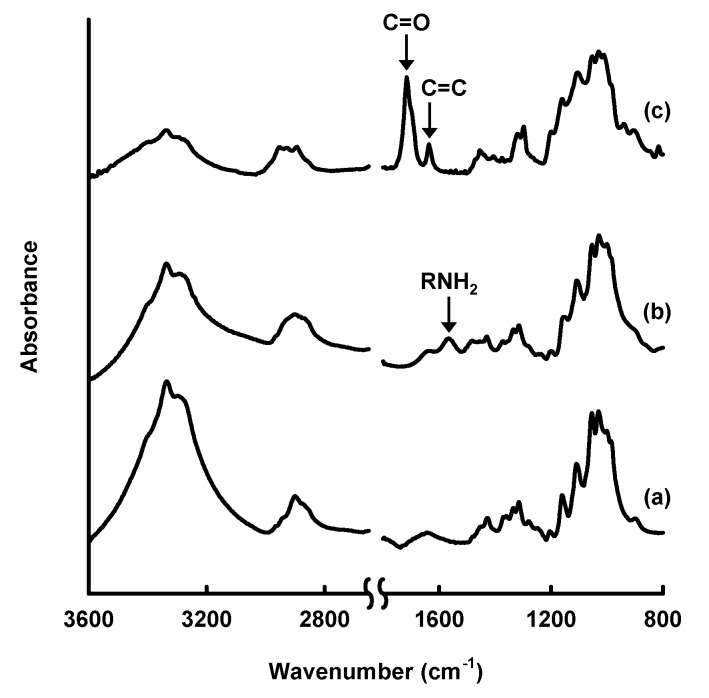
Fourier-transform infrared spectra of (a) original cellulose paper and cellulose paper treated with (b) 3-aminopropyltrimethoxysilane and (c) 3-(trimethoxysilyl)propyl methacrylate.

**Figure 4:**
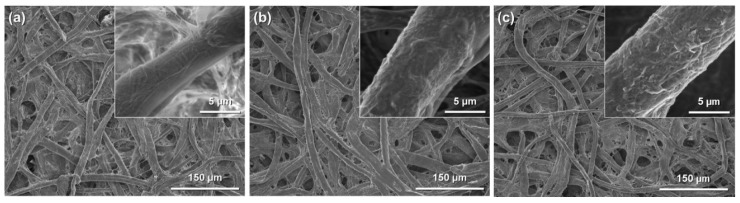
Field-emission scanning electron microscopy images of: (**a**) original paper; (**b**) amine-modified paper; and (**c**) methacrylate-modified paper.

In this review, the modification by silane coupling of cellulose in the form of paper with functional groups is described. Figure 2 shows a schematic diagram of the silane-coupling process on a cellulose paper substrate. The detailed experimental procedure has been reported in previous papers [33,34]. For example, amine-modified paper was prepared as follows. First, 3-aminopropyltrimethoxysilane was dissolved in a mixture of 80/20 (*v*/*v*) ethanol/water and, thus, hydrolyzed to form reactive silanol groups. Secondly, a piece of cellulose filter paper (cotton linter cellulose content: >99 wt %) was immersed in the obtained solution for 2 h, and then, the solvent was evaporated at 40 °C for 3 h under reduced pressure. Finally, the resulting paper was thermally treated at 110 °C for 3 h, followed by washing with ethanol and drying at room temperature. For the preparation of methacrylate-modified paper, 3-(trimethoxysilyl)propyl methacrylate and an aqueous acetic acid solution (pH = ~4) were used instead of 3-aminopropyltrimethoxysilane and ethanol/water. As shown in Figure 3, the cellulose papers treated with 3-aminopropyltrimethoxysilane and 3-(trimethoxysilyl)propyl methacrylate contained amino groups and methacryloxy groups, respectively, *i.e.*, the desired functional groups were successfully introduced *in situ* into the cellulose paper by a silane-coupling process, in which the condensation reaction between Si-OH groups of the silane-coupling agents and C-OH of cellulose forms Si-O-C bonds [31]. Figure 4 shows field-emission scanning electron microscopy images of the original, amine-modified and methacrylate-modified papers. The cellulose paper retained its specific interconnected porous microstructure, even after the silane-coupling treatment. 

However, changes in the surface morphology of the cellulose fibers were observed after modification with amino and methacryloxy groups, indicating that the silane-coupling agents were coated on their surfaces. The amounts of amino and methacryloxy groups introduced into the paper were estimated to be ~1.94 mmol·g^−1^ [33] and ~1.15 mmol·g^−1^ [34], respectively. The physical properties of the cellulose paper, such as the physical strength and hydrophobicity, were changed by the silane-coupling treatment. For example, the methacrylate-modified paper had a higher physical strength, including the Young’s modulus and tensile strength, than the original paper, especially in the wet state; the wet tensile strength of the methacrylate-modified paper (5.79 MPa) was more than seven-times higher than that of the original paper (0.76 MPa) [34]. This was possibly the result of the formation of bridges between cellulose fibers and the silane-coupling agents by cross-linking through condensation with the C-OH groups of cellulose and self-condensation between Si-OH groups. The hydrophobicity also improved significantly; the contact angle of water droplets on cellulose paper increased from 0° to ~110° after the introduction of methacryloxy groups [34], because of the introduction of hydrophobic moieties, *i.e.*, the propyl and methacryloxy groups of the silane-coupling agents, into the hydrophilic cellulose paper. The silane-coupling technique can be used to introduce polymers, such as polyethylenimine (PEI) [33], and various functional groups into cellulose paper and is a facile and useful technique for paper functionalization.

## 3. Amine-Modified Paper for Knoevenagel Condensation

Amino groups, which contain a basic nitrogen atom with a lone pair, have been widely used for a variety of processes, because of their many functions, including catalytic activity [35] and the ability to adsorb metal ions [36] and CO_2_ [37]. Amine-modified cellulose paper is therefore expected to have a wide range of applications. The Knoevenagel condensation between aldehydes or ketones and active methylene compounds [38] in the presence of a base catalyst is one of the important and convenient reactions for C-C bond formation [39,40]. Here, we describe the use of amine-modified paper as a base catalyst for the Knoevenagel condensation of benzaldehyde and ethyl cyanoacetate to produce α-cyanocinnamic acid ethyl ester [33], which is an important intermediate in the production of antihypertensive drugs. The Knoevenagel condensation was performed as follows [33]. Benzaldehyde (2.0 mmol) and ethyl cyanoacetate (1.7 mmol) were dissolved in an ethanol/water mixture (95/5, *v*/*v*; 10 mL). A piece of NH_2_- or PEI-modified paper (diameter 33 mm) was then immersed in the solution. The reaction was performed at 25 °C, with continuous stirring at 150 rpm. At a given time, the reaction solution was analyzed, using gas chromatography (GC), to determine the concentration of the desired product, α-cyanocinnamic acid ethyl ester. As controls, the reaction was also performed using the original cellulose paper, chitosan powder and liquid diethylamine.

**Figure 5:**
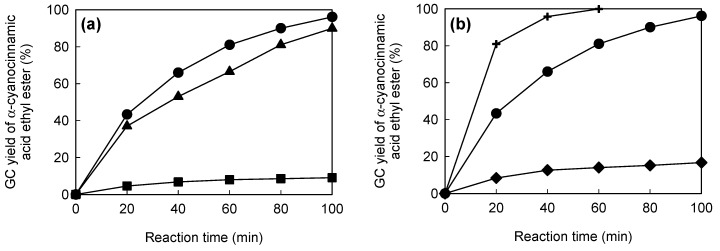
(**a**) Gas chromatographic (GC) yields of target product in batch mode: original paper (squares), NH_2_-modified paper (triangles) and polyethylenimine (PEI)-modified paper (circles). Amino group content: 0.00 mmol N (original paper), 0.20 mmol N (NH_2_-modified paper) and 0.54 mmol N (PEI-modified paper). (**b**) GC yields of the target product in batch mode: chitosan (diamonds), diethylamine (crosses) and PEI-modified paper (circles). Amino group content: 0.54 mmol N.

Figure 5a shows the catalytic performances of the original paper and the NH_2_- and PEI-modified papers, in batch mode. The original paper gave a poor catalytic performance, comparable to that in the absence of the paper, suggesting that cellulose paper had almost no catalytic activity in this reaction. In contrast, the NH_2_- and PEI-modified papers had much higher catalytic efficiencies than the original paper. These results indicate that amino groups introduced into the cellulose paper by silane coupling can act as a base catalyst. The PEI-modified paper had the best catalytic performance, because of its larger content of amino groups.

Figure 5b shows the catalytic performances of chitosan powder, liquid diethylamine and PEI-modified paper in batch mode. In all cases, the amino group content was set at 0.54 mmol of N. Chitosan, which is a basic polymer made from chitin, has been used as a base catalyst in several reactions, including the Knoevenagel condensation [41,42,43]. The PEI-modified paper had much higher catalytic efficiency than the chitosan powder. In the chitosan powder, amino groups are present not only on the surfaces, but also inside the crystals, resulting in poor contact between the reactants and the base sites. In contrast, the PEI-modified paper has a porous microstructure derived from the cellulose fiber networks (Figure 4), on which amino groups are exposed. This paper-specific feature contributes to the effective transport of the reactants to the base sites, resulting in the high catalytic performance of the PEI-modified paper. Liquid diethylamine gave the best performance, because the reactants and the base catalyst were in the same phase. However, it is difficult to reuse homogeneous catalysts. In contrast, the PEI-modified paper was recoverable after the reaction and was reusable without a significant decrease in the catalytic efficiency [33]. Amine-modified paper with a porous microstructure is therefore a promising and practical base catalyst for the Knoevenagel condensation.

## 4. Immobilized Lipase on Methacrylate-Modified Paper for Nonaqueous Transesterification

Enzymes, such as lipases (glycerol ester hydrolases E.C.3.1.1.3), are regarded as green catalysts, because of their good catalytic activities and selectivities under mild conditions [6,7]. Of many enzymatic reactions, lipase-catalyzed transesterification reactions in nonaqueous media have attracted much attention for use in the production of various useful chemicals through organic synthesis [44,45,46,47]. Enzymes, including lipases, are generally unstable in nonaqueous media and are easily aggregated and deactivated [48]; therefore, they are frequently immobilized on various supporting materials, such as silica [49,50], ceramics [51,52], carbonaceous materials [53] and polymers and resins [54,55], to provide reusability and stability in nonaqueous media [56,57] and to facilitate product isolation [57]. Lipases have relatively high hydrophobicity; therefore, simple adsorption of lipases on suitably hydrophobic supports though hydrophobic interactions is an effective immobilization approach [56,58,59]; ceramic supports modified with methacryloxy groups have been reported to be effective supporting materials for lipases [51,52]. The use of structured supporting materials has also been intensively investigated for the efficient and continuous production of target chemicals [22,52,60,61,62]. Here, we describe the immobilization of lipase on methacrylate-modified paper and the catalytic performance of the paper-immobilized enzyme in a nonaqueous transesterification reaction [34].

Lipase immobilization on cellulose paper was achieved by simply soaking the paper in a phosphate buffer solution of crude lipase powder [34]. Briefly, a piece of original or methacrylate-modified paper was soaked in the lipase solution, followed by stirring with an orbital platform shaker (23 °C for 12 h). The obtained paper was thoroughly washed with phosphate buffer solution at a stirring rate of 150 rpm for 3 h and dried in a desiccator at room temperature. The lipase content of the prepared paper was determined using a previously-reported colorimetric assay based on the hydrolysis of 4-nitrophenyl acetate [63,64]. The lipase immobilization yield on the methacrylate-modified paper was about 95%, whereas that on the original paper was about 30%. As described above, methacrylate-modified paper is hydrophobic; therefore lipases, which are relatively hydrophobic [58,59], were effectively attached to the hydrophobic surfaces of the methacrylate-modified paper by hydrophobic interactions. Modification of the cellulose paper with methacryloxy groups enabled efficient immobilization of lipase.

**Figure 6:**
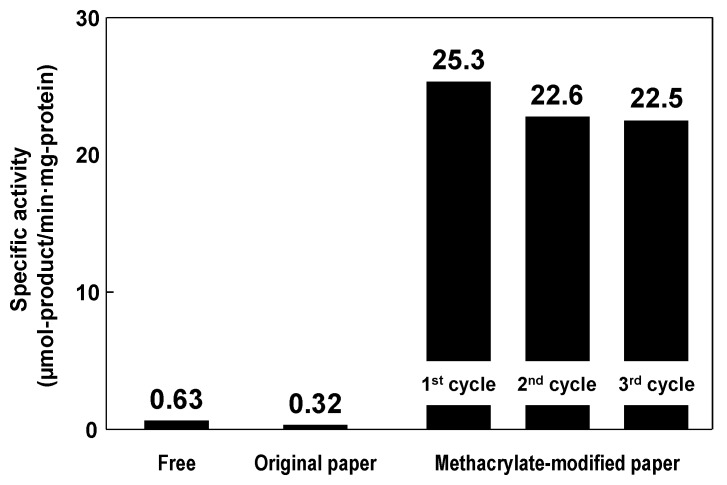
Specific activities of free lipase and lipase immobilized on original and methacrylate-modified papers. Reaction temperature: 23 °C. Stirring rate: 150 rpm.

The catalytic performance of the immobilized lipase on methacrylate-modified paper was evaluated in the nonaqueous transesterification reaction between 1-phenylethanol and vinyl acetate to produce 1-phenylethyl acetate, as follows [34]. In batch mode, a piece of original or methacrylate-modified paper (diameter 33 mm) with immobilized lipase or free lipase powder was immersed in an isopropyl ether solution (10 mL) of 1-phenylethanol (0.41 mmol) and vinyl acetate (0.62 mmol). The reaction was performed in a closed glass vial (diameter 40 mm, height 75 mm) at 23 °C, with and without continuous stirring by an orbital platform shaker. At a given time, the reaction solution was analyzed to determine the concentration of the target product, 1-phenylethyl acetate, by GC. Figure 6 shows the enzymatic activities of free lipase and of immobilized lipase on original and methacrylate-modified papers in batch-mode nonaqueous transesterifications. The specific activity of the immobilized lipase on methacrylate-modified paper was 25.3, which was 40-fold higher than that of the free lipase (0.63); the immobilized lipase on the original paper had poor specific activity (0.31), and the methacrylate-modified paper without lipase showed no catalytic activity in this reaction. Many researchers have reported that hydrophobic solid supports enable favorable interactions with the large hydrophobic pocket surrounding the catalytic sites of lipases, providing hyperactivation of lipases via interfacial activation, *i.e.*, the formation of a suitable open structure for effective contact with reactants [58,59]. It is therefore suggested that the hydrophobic methacrylate-modified paper support also contributes to the hyperactivation of lipases, leading to improved lipase activity in nonaqueous transesterification. The specific activity of the immobilized lipase on methacrylate-modified paper (25.3) was comparable with those on conventional supporting materials, including methacrylate-modified porous ceramics (37.2), diatomaceous earth (7.6), glass beads (0.8) and synthetic resins (0.4) [51]. In addition, the immobilized lipase on the methacrylate-modified paper was easily recovered after the performance test and was reusable without a significant decrease in its specific activity (Figure 6).

**Figure 7:**
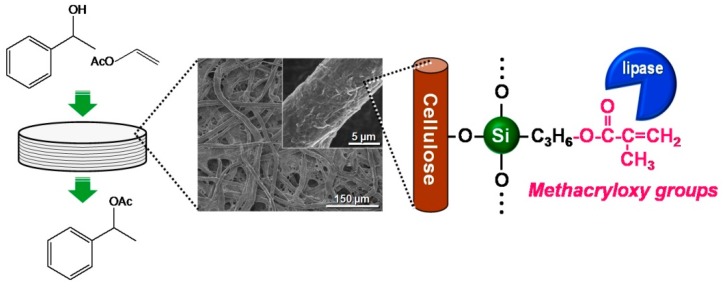
Schematic diagram of the continuous-flow enzymatic reaction using lipase immobilized on methacrylate-modified paper.

The advantages of cellulose papers as supporting materials over conventional materials include applicability to flow processes for the continuous synthesis of target products, as a result of their highly porous structure. Continuous-flow catalytic reactions using lipase immobilized on methacrylate-modified paper was therefore investigated (Figure 7). For the flow-type reaction, immobilized lipase on methacrylate-modified paper was cut into 14 circular discs (diameter 9.0 mm), vertically stacked (thickness ~3.1 mm) and then tightly packed into a syringe equipped with a silicon tube.

**Figure 8:**
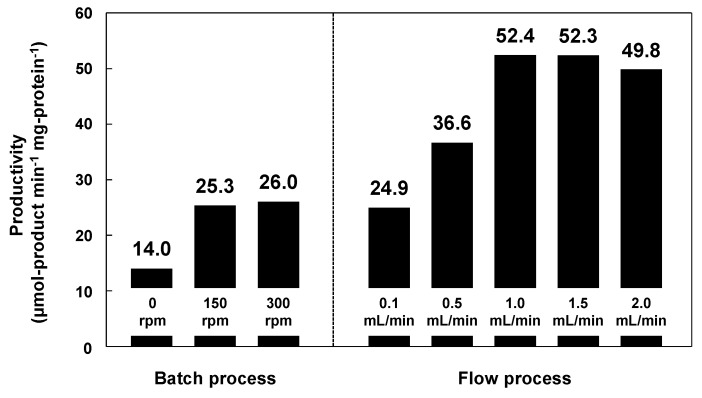
Production of 1-phenylethyl acetate in batch and flow processes using lipase immobilized on methacrylate-modified paper. The lipase content was equal in each case. Reaction temperature: 23 °C. Stirring rates in batch mode: 0, 150 and 300 rpm. Flow rates in flow mode: 0.1, 0.5, 1.0, 1.5 and 2.0 mL·min^−1^.

Subsequently, the reactant solution was fed into the paper layer at a constant flow rate of 0.1–2.0 mL·min^−1^ using a syringe pump. Figure 8 shows the amounts of 1-phenylethyl acetate produced in batch and flow modes. The productivity using lipase immobilized on methacrylate-modified paper was higher in flow mode than in batch mode; the maximum productivity was recorded at a flow rate of 1.0 mL·min^−^^1^ (52.3), which was approximately double that in batch mode at a stirring rate of 300 rpm (26.0). In the batch processes, which were performed in closed glass vials (diameter: 40 mm; height: 75 mm), the diffusion length of the reactants was millimeter scale, resulting in relatively low productivity, because the accessibility of the reactants to the lipase on the paper was insufficient, even under vigorous stirring. In contrast, the flow reaction proceeded through the interconnected micrometer-scale pores of the paper. The short diffusion paths inside the paper provide fast mixing of the reactants and effective transport of the reactants to the catalytically-active sites of the lipase, leading to high productivity. Such an interesting effect has also been reported for the metal catalysts/ceramic paper composites in various catalytic applications [65,66,67,68,69,70]; the paper-structured supports were more effective than the conventional honeycomb-structured supports, possibly due to the paper-specific interconnected porous microstructures [68]. Cellulose paper therefore has potential applications in a new class of microstructured reactors with excellent practical properties, and paper-immobilized enzymes are promising catalytic materials for the continuous production of useful chemicals.

## 5. Conclusions

In this review article, we describe the *in situ* modification of cellulose paper with various functional groups, using a silane-coupling technique, and the use of the prepared, chemically-modified papers as microstructured catalytic reactors for the effective production of useful chemicals. The chemically-modified catalytic paper can be easily reused and can fit various reactor configurations. Furthermore, the paper-specific interconnected porous microstructure provides higher productivity in continuous-flow reaction systems than in batch reactions, possibly through the provision of favorable diffusion paths for the reactants. The additional advantages of cellulose paper over other supporting materials include sustainability and high mass production. Cellulose is highly stable in most solvents and is both hydrophilic and lipophilic, giving it advantages for use in various reaction systems. Because cellulose is thermally degraded around 300 °C, chemically-modified paper can be useful in relatively low-temperature catalytic processes, such as liquid-phase reactions. The structural design of the paper would be effective for further improvement of the catalytic efficiency to promote its practical implementation. Thus, chemically-modified cellulose paper is a green microstructured supporting material that has excellent practical properties and provides favorable reaction fields for various catalysts. These findings open new doors for green and sustainable catalytic processes in future chemical industries.
